# Temperature Assessment Of Microwave-Enhanced Heating Processes

**DOI:** 10.1038/s41598-019-47296-0

**Published:** 2019-07-25

**Authors:** B. García-Baños, J. J. Reinosa, F. L. Peñaranda-Foix, J. F. Fernández, J. M. Catalá-Civera

**Affiliations:** 1 ITACA Institute, Universitat Politècnica de València, Valencia, 46022 Spain; 2 Instituto de Cerámica y Vidrio, CSIC, Madrid, 28049 Spain

**Keywords:** Electrical and electronic engineering, Characterization and analytical techniques

## Abstract

In this study, real-time and *in-situ* permittivity measurements under intense microwave electromagnetic fields are proposed as a powerful technique for the study of microwave-enhanced thermal processes in materials. In order to draw reliable conclusions about the temperatures at which transformations occur, we address how to accurately measure the bulk temperature of the samples under microwave irradiation. A new temperature calibration method merging data from four independent techniques is developed to obtain the bulk temperature as a function of the surface temperature in thermal processes under microwave conditions. Additionally, other analysis techniques such as Differential Thermal Analysis (DTA) or Raman spectroscopy are correlated to dielectric permittivity measurements and the temperatures of thermal transitions observed using each technique are compared. Our findings reveal that the combination of all these procedures could help prove the existence of specific non-thermal microwave effects in a scientifically meaningful way.

## Introduction

For many years, microwave energy has been increasingly and successfully used to process materials with unique properties at high temperatures (>1000 °C) in a broad range of traditional and emerging manufacturing fields of materials such as ceramics, metals or in nanotechnology.

The benefits of using microwaves for heating materials are well-known and have been widely recognized over the last decades. In microwave processing, energy is rapidly transformed into heat inside the materials through electromagnetic (EM) radiation, whereas in conventional heating, thermal energy is merely propagated throughout the volume of the specimen by an external heating source.

Numerous studies on microwave processing have revealed that microwave technology significantly reduces reaction time and influences material properties^1–7^. Moreover, an important decrease has been reported in the reaction temperature of hundreds of degrees centigrade in comparison to conventional heating means^1^ which was associated to a substantial decrease in the apparent activation energy.

There is a constant debate, however, on whether the observed rate enhancement attained with this ultra-fast microwave dielectric heating is caused by purely thermal effects, or if this is connected to specific or non-thermal microwave effects arising from the interactions between the radiation fields and the material^1,6,8–12^. While there is no doubt that thermal microwave effects do exist, the specific or non-thermal microwave effects are more difficult to justify and are subject to constant speculation and discussion, as many previously reported non-thermal microwave effects have turned out to be purely thermal in nature^8^. Solving this significant yet basic problem is thus a major challenge for researchers in the field of microwave processing.

Exploiting the many facets of microwave processing requires a thorough and comprehensive understanding of the underlying processes at atomic level for which new and complex instruments and measurement capabilities are needed. In this regard, several approaches have been developed for the *in-situ* monitoring of microwave reactions in combination with other characterization techniques for solid materials as X-Ray Diffraction, Neutron Scattering, FTIR or Raman Spectroscopy^13–16^. Schmink and Leadbeater^15^ and Vaucher *et al*.^16^ employed *in-situ* Raman spectrometers to examine heating selectivity during microwave exposure. However, although they are ground-breaking studies, additional experimental evidence is still required.

The interaction between electromagnetic waves and matter is quantified by complex physical parameters such as electrical conductivity and dielectric properties (or permittivity). These interactions depend on the structure of the materials and chemical bonds between their atoms as electromagnetic fields couple directly to the electrons in atoms, molecules and crystals or cluster structures of materials. Hence, it has also been suggested that the measurement of dielectric properties and the variations of this measurement with other process-related parameters (temperature, phase transitions, etc.) could shed light on the thermal processes of materials under microwave fields^10,12,17–19^.

There is a wide range of reported experimental techniques to determine the dielectric properties of materials as a function of temperature from very low frequencies to several hundred gigahertzes (a brief overview of these methods can be found^18^). For most of the permittivity experiments in the GHz range, especially around the Industrial Scientific and Medical (ISM) frequencies 0.915 GHz and 2.45 GHz, the thermal process was carried out by increasing the temperature of the materials using conventional means (infrared, electric) to moderate temperatures (200 °C to 500 °C), which does not allow us to observe microwave effects with the monitoring of permittivity changes with temperature.

As to experimental research into microwave applications, the *in-situ* and real-time observation of dielectric properties of materials during microwave heating, together with the synchronous recording of other process parameters, recently described by Catala-Civera *et al*.^20^ has provided the basis for a better understanding of the interaction of materials with electromagnetic fields.

However, there are three particular issues to be dealt with in order to further develop these techniques: (i) the ultrafast nature of microwave reactions; (ii) the compatibility of experimental methods with microwave technology; and (iii) the complexity of the temperature measurement procedures.

In order to demonstrate the existence of the above mentioned specific microwave effects, the bulk temperature reached during the microwave reaction process must be determined accurately. The vast majority of experiments conducted under microwave conditions have demonstrated that what controls the outcome of a chemical reaction is solely the bulk temperature in the material^8^.

The precise measurement of reaction temperature is a non-trivial and complex matter which entails basic knowledge of the effects caused by microwave dielectric heating^21–24^. In the past, the reaction temperatures under microwave heating conditions were very often measured inappropriately which led to many misguided claims of certain non-thermal microwave effects. When the experiments were repeated using adequate temperature monitoring and controls, it was proven that some of the originally proposed specific non-thermal effects were simply thermal in nature^21^.

Apart from the singularities of the microwave heating process, what makes measuring reaction temperatures under microwave conditions such a complicated task is the inconvenience of using classical temperature measurement instruments such as thermocouples, since these devices are metallic and will couple with the EM fields. Also, as fiber-optic sensors are extremely fragile, the sample temperature is usually measured with an IR pyrometer pointing at the sample surface from outside the microwave heating chamber^19–24^. There are also reasonable doubts about the accuracy of temperature measurements, given that microwave heating can lead to important temperature gradients between the center and the surface of the sample, which increase particularly at high temperatures.

Thus, determining the real bulk temperature during the microwave irradiation could allow us to verify whether specific non-thermal microwave effects actually exist in a scientifically robust way.

In this study, a new temperature calibration method merging data from four independent techniques is developed to obtain the bulk temperature as a function of the surface temperature. We propose real-time and *in-situ* permittivity measurements under intense microwave electromagnetic fields together with the simultaneous determination of dielectric properties and synchronous recording of some thermo-physical and mechanical properties as a powerful technique for the study of thermal processes in materials.

In order to draw reliable conclusions from the temperatures at which the transformations occur, we first address the accurate measurement of the bulk temperature under microwave conditions. Also carried out are comparative permittivity measurements with other analysis techniques such as Differential Thermal Analysis (DTA) or Raman spectroscopy to contrast thermal transitions with some selected materials.

Our findings allow for deeper theoretical insight into the high-temperature ultrafast interaction of materials with microwaves and its kinetics, and most importantly, provide a solid experimental basis which, for the first time, could assess the existence of thermal or non-thermal effects in upcoming microwave heating experiments leading to new developments in microwave technology.

## Results and Discussion

### Experimental setup

**Figure 1:**
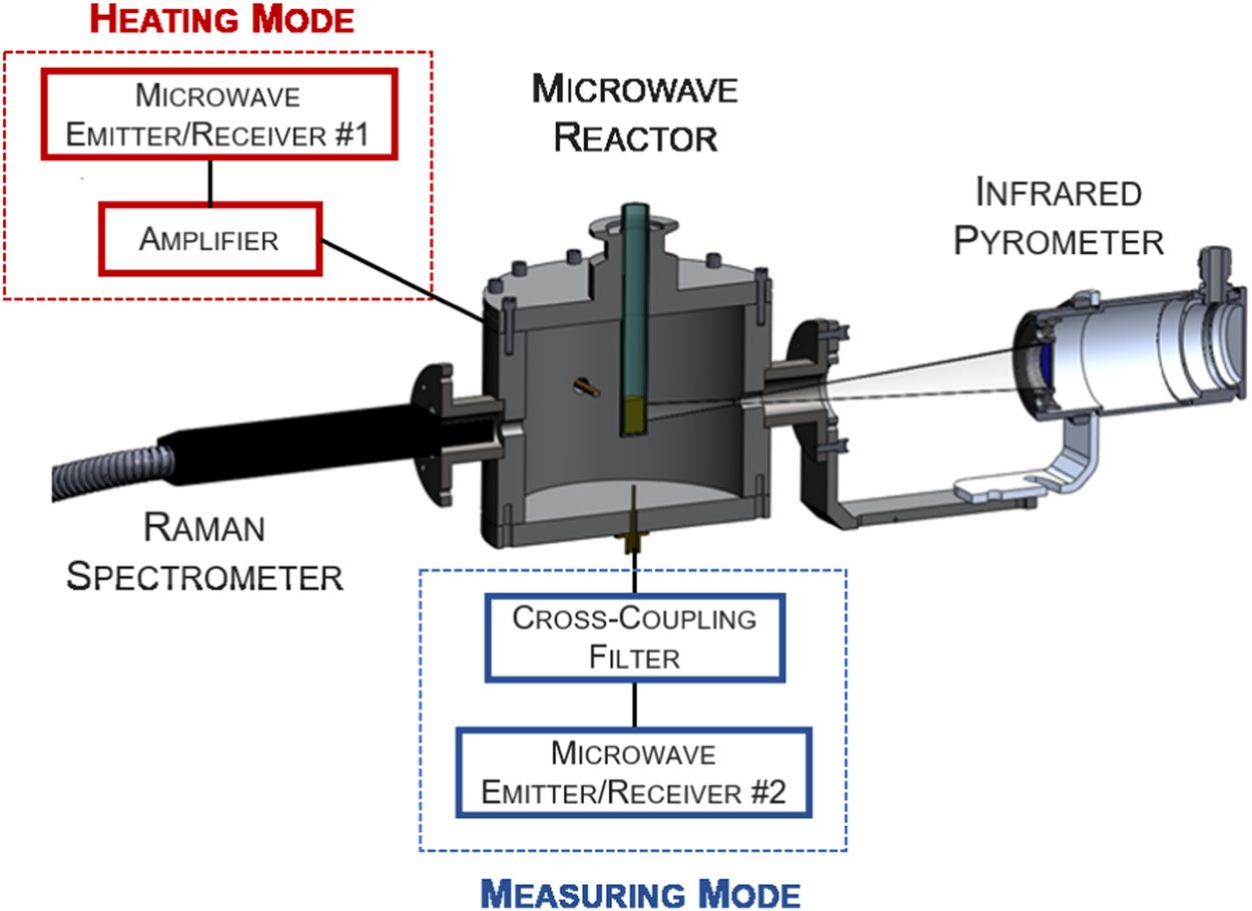
Experimental Setup. Cylindrical reactor for microwave heating and dielectric measurements together with the IR temperature and Raman sensors for *in-situ* material inspection during microwave exposure.

The experimental setup (Fig. 1) used in our study for microwave heating and *in-situ* permittivity measurements of dielectric samples consisted of a dual-mode microwave cylindrical cavity capable to heat and measure with two separate microwave systems without interferences. The first of these systems, used for the heating process and coupled to the cavity by the horizontal probe located on the back sidewall of the cavity, delivered a microwave power signal (~150 W) around the ISM frequency of 2.45 GHz (from 2.4 to 2.5 GHz), while the second system, coupled to the vertical probe located at the bottom wall, generated low power microwaves between 1.8 and 2.2 GHz to characterize the dielectric properties of the materials by the Cavity Perturbation Method (see Methods section).

Samples of dielectric materials were prepared in a cylindrical shape (10 mm diameter, 15 mm height) to fit inside the quartz vials (1 mm wall thickness) which were inserted into the center of the cavity through a hole located on the top wall. The reactor was designed to ensure a strong and uniform electric field distribution in the cavity where the sample was positioned during both operation modes (heating and measuring). Quartz holders were employed as they are transparent to microwave radiation and can withstand temperatures above 1000 °C.

Figure 1 also shows the access holes on the sidewall of the reactor which allow sample inspection during the microwave heating process. An infrared (IR) pyrometer (Optris CT-Laser LT) was used to measure the surface temperature of the quartz holder from the outside of one of these holes, and a portable Raman spectrometer (ProRaman-L-532-B1S) was installed in another access hole to observe *in-situ* structural transformations. In addition, a video camera was installed in a third hole (not visible in Fig. 1) to record the changes in shape during processing. Every access was designed to prevent the leakage of microwave energy outside the cavity and to avoid interferences in the measurement procedures. More details about the setup can be found^20^.

### Temperature Calibration

The previously described experimental setup has, however, a few limitations. One of them is that the low thermal conductivity of the quartz tubes can delay the detection of thermal effects in the samples. The second, and most important limitation, comes from the fact that, although the microwave reactor was designed to generate heat uniformly, the air surrounding the sample remains cool at room temperature which causes a decrease in the surface temperature of the sample leading to the well-known inverted temperature gradients of microwave heating that are not found in conventional heating. Thus, the measurement of the surface temperature of the quartz holder provided by the IR pyrometer, as shown in Fig. 1, cannot be considered as representative of the sample temperature and a new calibration procedure is required to determine the bulk temperature of the sample, at which the reactions takes place.

In this study, we propose a calibration procedure to determine the average value of the bulk temperature of a dielectric sample heated by microwave energy. The mean value of the sample temperature is obtained as a function of the surface temperature of the holder measured by the IR pyrometer. To minimize the errors and uncertainties that would arise from a single calibration method, four different independent calibration techniques are developed. We utilize the described equipment together with a conventional heater, fiber optic sensors, a Raman spectrometer and pure salts. The various techniques are described in the following subsections.

### A. Calibration through conventional heating

The temperature calibration with a conventional heater consisted in inserting a cartridge heater with controllable temperature inside the quartz tube of the microwave cavity and then comparing its temperature with that of the tube’s surface measured by the IR thermometer.

**Figure 2:**
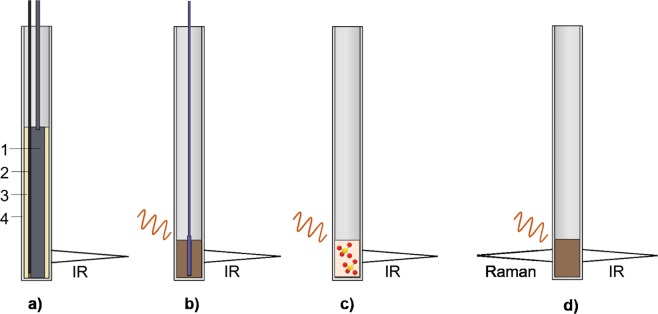
Schematic view of the sample in the different calibration techniques: **a**) using conventional heating (1-cartridge heater, 2-thermocouple, 3-sample tube, 4-quartz holder); **b**) with fiber optic sensor; **c**) using calibration salts and **d**) with Raman spectrometer.

To incorporate the effect of temperature gradients from the core of the sample to the surface of the sample and container which dielectric samples of different composition might show, the cartridge heater was also surrounded by a hollow cylinder of a second material with different thermal conductivity which filled the space between it and the quartz holder (see Fig. 2a). Materials ranging from highly insulating such as alumina (Al_2_O_3_) to highly conductive like aluminium were employed to encase the heater during the experimental work.

**Figure 3:**
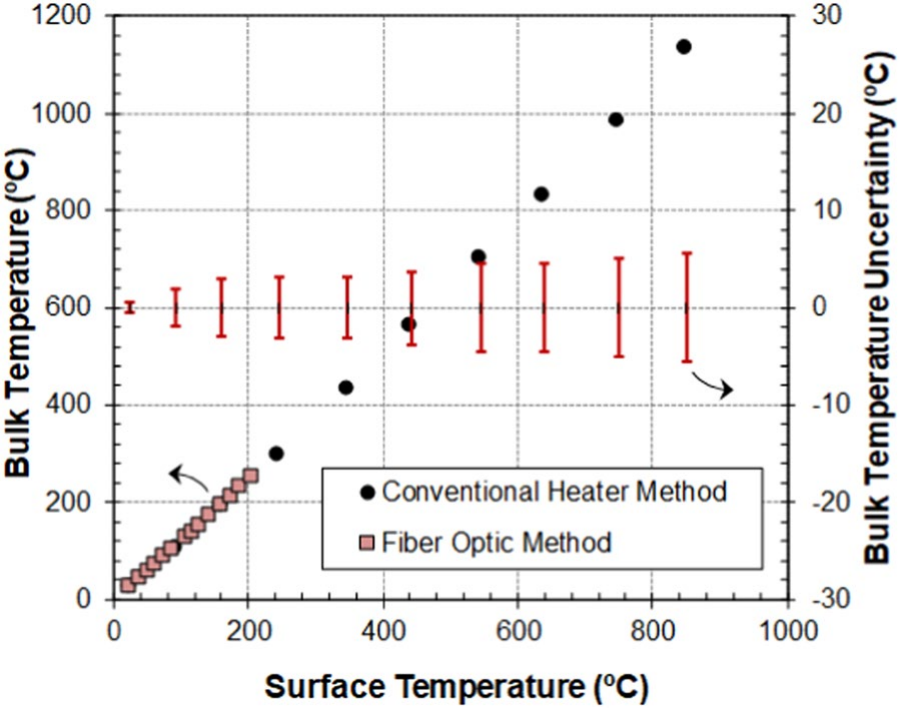
Temperature calibration curve obtained with two calibration techniques: using conventional heating (calibration materials: alumina, distilled water, silicon carbide and aluminium) and with fiber optic (FO) sensor respectively.

As the microwaves were switched off during the calibration procedure, we were able to use a metallic thermocouple capable of reading temperatures over 1000 °C to measure the temperature of the cartridge heater core. Figure 3 shows the average temperature of two extreme materials of different conductivity given by the thermocouple sensor as a function of the surface temperature measured from outside by the IR thermometer. A heating rate of 1 °C/min was fixed for all heating experiments.

The uncertainty range in the determination of the bulk temperature from the measurement of the holder surface temperature is also represented in Fig. 3 (right axis). It refers to the maximum difference observed in the bulk temperature between the different materials for a given surface temperature. This range is below ± 5.5 °C in the bulk temperature range from 22 °C to 1100 °C, which is a percentage lower than 2%.

### B. Calibration with Fiber Optic Sensor

Fiber optic (FO) sensors represent an accurate alternative to determine the temperature of bodies under microwave heating by placing them in direct contact with the material of interest. Unlike thermocouples, FO measurement devices are immune to electromagnetic interferences and do not require shielding. However, FO probes are extremely fragile and their main limitation, as compared to IR thermometers is that their narrow temperature measurement range normally only reaches up to 300 °C^21^.

The FO calibration procedure consisted in heating a sample within the microwave cavity described in Fig. 1 and comparing the bulk temperature measured by the FO with the surface temperature given by the IR pyrometer (Fig. 2b).

The materials selected for this technique were distilled water and silicon carbide (SiC), which have good microwave absorbing properties and dissimilar thermal conductivities. Samples of these materials were poured into the quartz container to a height of 15 mm and subjected to microwave heating. The microwave power was adjusted at an approximate rate of 5 °C/min.

The ratio between the measured surface and FO bulk temperature is also plotted in Fig. 3. Our results with this procedure are in good agreement with the relationship found in the previous section.

### C. Calibration with pure salts

The temperature calibration procedure described in this section consisted in using the information given by the continuous measurement of dielectric properties as a function of the surface temperature during microwave heating to identify thermal phenomena in some reference materials with well-known transition bulk temperatures (Fig. 2c). For this procedure we selected pure salts, which are commonly used for temperature calibration in thermal analysis devices (e.g. DTA).

For our first experiment we chose silver sulfate (Ag_2_SO_4_), which undergoes a change in its crystalline structure from an orthorhombic configuration to a hexagonal one at 425 ± 4.8 °C, according to bibliography^25–30^. A sample of this salt was poured into the quartz tube. It was then heated by microwave irradiation in the microwave cavity shown in Fig. 1, adjusting the heating power at an approximate rate of 5 °C/min.

**Figure 4:**
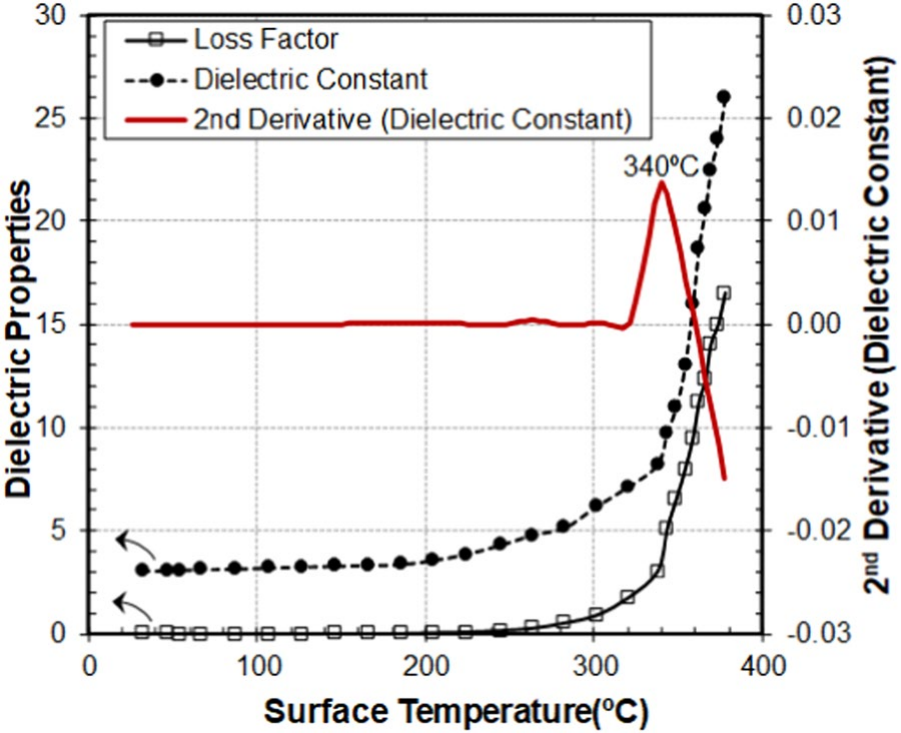
Dielectric properties (Dielectric constant and loss factor) of silver sulfate as a function of the holder surface temperature measured by the pyrometer. The second derivative of the dielectric constant facilitates the identification of the temperature at which the sample changes its crystalline structure.

Figure 4 illustrates the measured dielectric properties (dielectric constant and loss factor) of silver sulfate as a function of the holder surface temperature. As can be observed, the dielectric properties show a progressive increase with temperature to approximately 300 °C where a fast change takes place. Previous studies^29^ showed that the change from *β-*Ag2SO4 to *α-*Ag2SO4 leads to a different deformability of the Ag + ion electron cloud, increasing the polarizability of the Ag + ion. This is consistent with the observed higher dielectric constant after the transition.

To better identify the transition temperatures around the changes in the salt, we made use of a second derivate that emphasized the variations of the dielectric properties around the measured surface temperature of 340 °C, which we assume corresponds to the phase transformation at a bulk temperature of 425 °C in the sample.

To further determine temperature calibration points, we experimented with another salt: potassium perchlorate (KClO_4_). This material undergoes a sharp orthorhombic to cubic phase transition at a bulk temperature of 298.9 ± 0.9 °C^26,27,29,30^. The phase transition was also identified by a strong change in the dielectric properties when the pyrometer indicated a surface temperature of 245 °C (see details in supplementary Fig. S1).

This technique is valid for other salts and materials whose thermal transformations are visible by dielectric measurements.

### D. Calibration with A Raman spectrometer

Following a similar procedure as before to identify thermal effects this time we used reference materials with well-known Raman shifts. We first selected bismuth titanate (Bi_4_Ti_3_O_12_) which belongs to the Aurivillius family and shows ferroelectric and piezoelectric properties up to high temperatures. Its Raman response reflects an orthorhombic to tetragonal phase transition at a bulk temperature of 677.5 °C ± 2.5 °C^31–33^.

**Figure 5:**
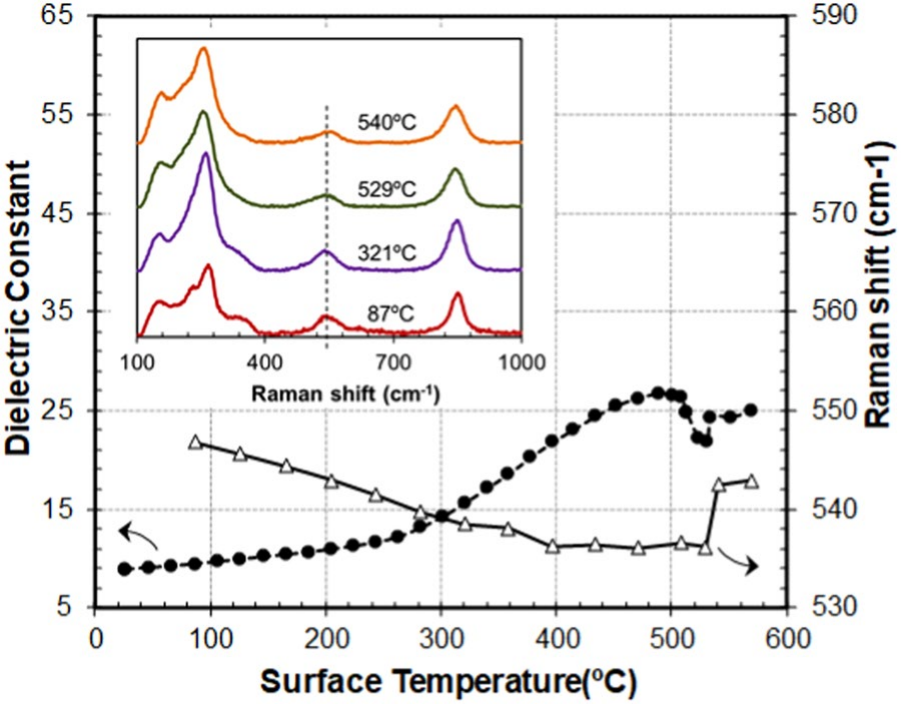
Dielectric constant and Raman shift of the mode placed around 500–650 cm^−1^ during microwave heating of a bismuth titanate sample. Inset: Raman shift spectrums obtained at different temperatures during the heating process (the dashed line shows the Raman mode of interest).

A sample of bismuth titanate was placed in the quartz holder and subjected to microwave heating in the reactor (Fig. 2d). The heating rate was fixed at 10 °C/min. Figure 5 illustrates the variation of the dielectric properties of the sample and compares the holder surface temperature with the Raman shift. Close to the transition temperature, there is a decrease of the dielectric constant related to an arrangement of the structure^32^. In particular, this perovskite structure presents 3 octahedrons made of TiO_6_ units. These octahedrons are linked by apical oxygen atoms and, initially, the octahedrons present displacement between them. When the temperature increases, the octahedrons stay in the same axis, eliminate the displacement, the structure modifies its behavior from ferroelectric to paraelectric and the polarizability of the structure decreases. As a result, the dielectric constant also decreases.

The transition was evidenced by the behavior of the Raman mode around 500–650 cm^−1^, which correlates fairly well with other studies^31^. The inset shows some of the Raman spectrums recorded at different temperatures during the experiment. The dielectric and Raman spectroscopy measurements and their comparison in Fig. 5 allowed us to identify the surface temperature of the structural change at 529.5 °C which is assumed to reflect the bulk material temperature of 677.5 °C.

Next we applied a similar technique to a sample of potassium sulfate (K_2_SO_4_). This salt undergoes a change in its crystal structure from an orthorhombic to a hexagonal system at a bulk temperature of 585 ± 2 °C^26,27,29,34^. The Raman response of the mode around 980 cm^−1^ experienced a sharp shift at a holder surface temperature of 463 °C, which was correlated with the dielectric measurements (see details in supplementary Fig. S2).

**Figure 6:**
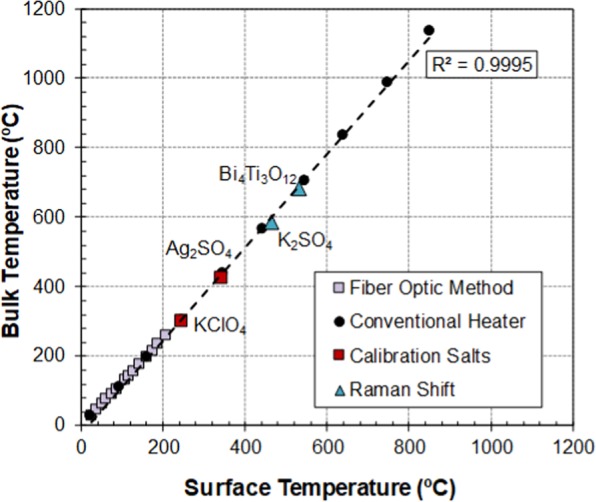
Temperature calibration curve obtained with the combination of the four techniques.

Finally, all the temperature calibration results of the above described procedures were merged together into one single graph (Fig. 6). As can be appreciated, the results obtained from the different techniques are consistent and fall in the same straight line. Thus, the application of any of the four, or a combination of them, could be used as a reliable method for estimating the bulk temperature of samples in this microwave system. As expected, the calibration curve shows that the difference between surface and bulk temperature gradually increases at higher temperatures, showing that non-adequate calibration of this difference could lead to errors in the bulk temperature determination of the order of 200 °C at high temperatures. It should be noted that the maximum deviation between the measurements and the represented calibration curve (dashed line in Fig. 6) is only ± 4 °C in the range from 20 °C to 1000 °C.

### Demonstration

In order to obtain reliable information about the influence of microwave fields on heating mechanisms, the calibration procedures described above were applied to study thermal phenomena in two different materials.

The first material chosen for our demonstration was the Standard Reference Material 717 A provided by NIST (National Institute of Standards and Technology in Boulder, Colorado), a borosilicate glass known for its interesting physical and chemical features and commonly used to calibrate equipment for testing viscosity and other glass properties in accordance with ASTM procedure C 965–81^35^.

Annealing and strain temperatures are both thermal parameters directly related to the stress in the molecular structure of glass and have a direct influence on the variation of viscosity of this material at these temperatures. Some studies that have measured the electric properties of glass have revealed that the viscosity of this material can be directly correlated with its internal structure. As the glass temperature rises, there is an increase in the polarization of atomic bonds and in the bonding distance between atoms, which means that there is a continuous increase in the dielectric constant, while the viscosity of the sample diminishes^36–38^.

The strain and annealing temperatures of 717 A glass, certified by NIST and several other laboratories^35^, are 470 ± 9 °C and 513 ± 6 °C, respectively.

To measure the variation of the dielectric properties of borosilicate glass under microwave irradiation, we produced a cylindrical rod 10 mm wide and 15 mm high which was inserted into the quartz vial at the cavity center. As expected, the changes in glass viscosity due to the increasing temperature led to perceptible changes in the dielectric constant.

**Figure 7:**
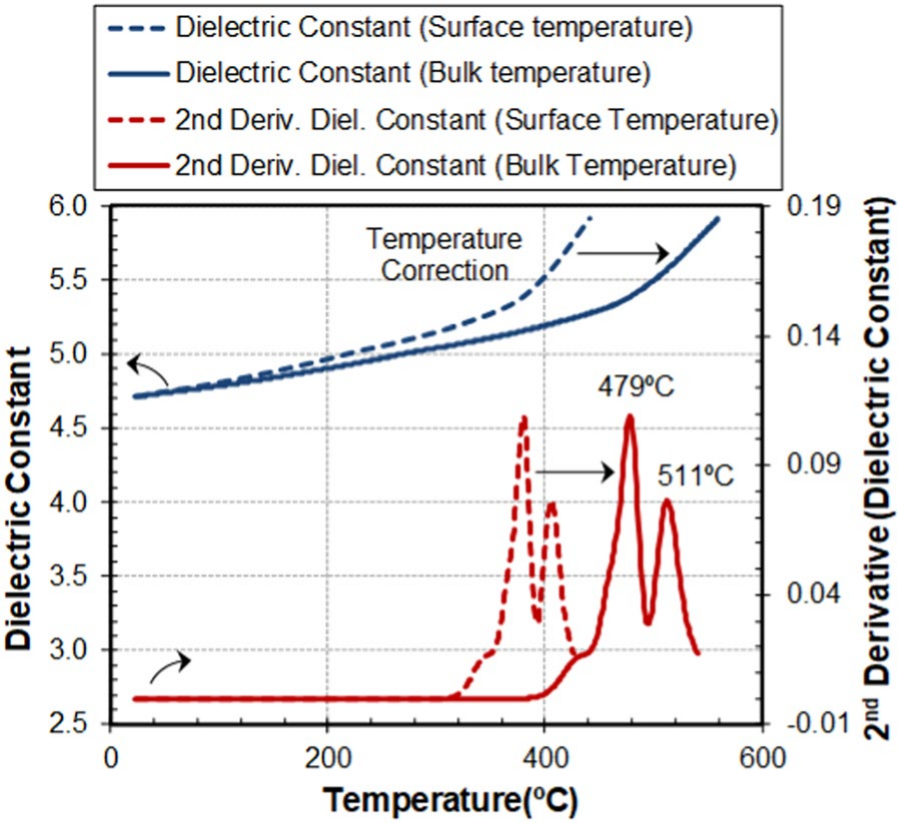
Dielectric constant of borosilicate glass 717 A and second derivative vs. temperature. Dashed lines represent the measurements vs. the surface temperature. Solid lines represent the measurements vs. the bulk temperature of the sample, after temperature calibration. Peaks in the second derivative correspond to the strain and annealing temperatures.

Figure 7 plots the measured dielectric constant of the 717 A sample as a function of the temperature that shows a steady variation up to approximately 450 °C following which there is a clear increase in the slope indicating material changes around this temperature. The dashed lines show the dielectric measurement of the holder surface temperature whereas the solid lines correspond to that of the sample bulk temperature, after calibration.

A second derivative of the dielectric constant was employed to allow a rapid identification of the temperatures at which the growth tendency changes. The peak temperatures (479 °C and 511 °C), visible in Fig. 7, correlate well with the strain and annealing temperatures certified by NIST for this glass composition. Furthermore, the good correspondence between the strain and annealing temperatures of this glass under conventional and microwave heating suggests that there are no specific effects caused by the application of microwave fields.

Based on these results, the measurement of dielectric properties as a function of the temperature at microwave frequencies can be also considered as a suitable methodology to determine thermal parameters of interest in glass, such as strain and annealing temperatures, especially for those types of glass that present limitations when more common measurement procedures at lower frequency ranges (in the KHz-MHz) are employed. These measurement problems are due to the reduced sensitivity since viscosity can be masked by conductivity at low frequencies^39–41^.

For a second microwave experiment we used a ceramic frit composed of a mixture of raw materials (quartz, feldspars, calcium carbonate and zinc oxide), which undergoes phase transformations at high temperatures.

A sample was placed in the quartz container, inside the microwave reactor, and heated to more than 1000 °C. In addition to measuring this material’s dielectric properties, a DTA measurement was carried out separately for the comparison of the phase transformations and transition temperatures.

**Figure 8:**
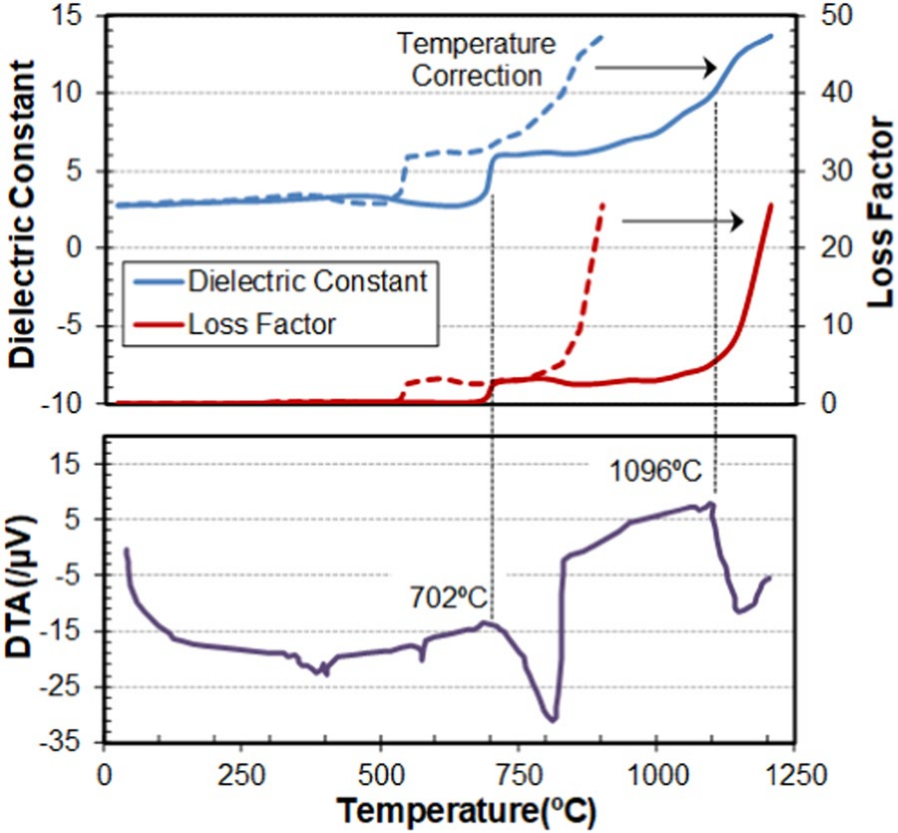
Dielectric properties (Dielectric constant and Loss factor) and Differential Thermal Analysis (DTA) of ceramic frit raw materials vs. temperature. Solid lines represent the measurements vs. the bulk temperature of the sample, after temperature calibration.

Figure 8 shows the variation of the dielectric properties as a function of the temperature, before and after temperature calibration, as well as the results of the DTA measurement for comparison. As Fig. 8 illustrates, there were no significant variations in the dielectric properties of the frit sample until approximately 700 °C, where a first sharp transition occurred together with a rapid increase in the sample temperature.

Although different mechanisms have been attributed to this process, practically all of them show the existence of alkaline-earth cation diffusion in the sample up to achieve the complete de-carbonation of the sample^42^. Moreover, the increase of dielectric properties of the frit sample could be caused by the movement of charges in the sample until the accommodation of the final structure during the dolomite decarbonation process.

In addition, a second change is observed in the dielectric properties response around 1100 °C, especially noticeable in the loss factor, which may be explained by the change in the viscosity of the sample due to melting. It should be noted that in changes of state at high temperature there is an initial increase of the atomic bond distances and, in some cases, an atomic breakdown. Furthermore, in the case of ceramic frit, the mixture of different raw materials triggers chemical reactions and mass transport in the sample. Hence, in our case, the variations in the dielectric properties cannot be attributed to a single cause but to all the above mentioned ones.

The DTA measurement revealed a first transition at approximately 700 °C, which is related to the dolomite decarbonation together with an endothermic reaction, and a second one around 1100 °C associated to the frit melting.

As in the first experiment, after temperature corrections, the transition temperatures identified by the dielectric measurements under microwave heating in both thermal phenomena of the ceramic frit were in good agreement with the results obtained by conventional techniques as DTA.

Again, the material studied in the second microwave heating experiment underwent similar transformations to those which occur under conventional heating and no special mechanisms were evidenced due to the presence of the microwave fields.

Several studies have recently reported that microwave heating could trigger transformations in materials at temperatures several hundred degrees centigrade lower than under conventional heating, leading them to claim that non-thermal or specific microwave effects do exist. Nevertheless, our findings revealed that differences between the bulk temperature and the surface temperature of the sample can be of the order of several hundred degrees. Consequently, there are reasonable doubts about the accuracy of the temperature measurement procedures employed in these studies, which needs to be corrected.

The temperature calibration during microwave heating in our system has been achieved, among others, by measuring materials with phase changes. In the phase transition, structural changes occur in the solids at fixed temperatures that entail changes in their polarizability. Despite of the fact that the calibration procedure made use of solid materials, the calibration line defines the real bulk temperature of a material during the heating process using our recently developed microwave reactor described here and it can be extended to liquids, solids and their mixtures. The results presented open a field of work that allows discriminating accurately the different phenomena during the heating with microwave energy, eliminating the uncertainty about temperature. Considering the above findings, we believe that the new approach to calibrate temperature measurements in our microwave reactor serves not only to assess the bulk temperature of materials when heated by microwaves, but can also be used as a basis for research about thermal and non-thermal effects under microwave irradiation in upcoming experiments.

## Methods

The samples employed in the measurements are summarized in Table 1:

**Table 1:** Samples employed in the experiments.

Material	Supplier	CAS No.	Format	Purity	Weight (g)	Density (g/cm^3^)
Al_2_O_3_	Nanoker	1344–28–1	Tube	99.7%	-	-
SiC (F100)	ABCR	409–21–2	Powder	≥99.9%	2.17	1.92
Distilled water	Panreac	7732–18–5	Liquid	-	1.09	1
Ag_2_SO_4_	Sigma-Aldrich	10294–26–5	Powder	≥99.5%	3.10	2.74
KClO_4_	Sigma-Aldrich	7778–74–7	Powder	≥99.0%	1.86	1.64
Bi_4_Ti_3_O_12_	Prepared at CSIC	—	Powder	—	3.34	2.95
K_2_SO_4_	Sigma-Aldrich	7778–80–5	Powder	≥99.0%	2.10	1.86
Glass 717 A	NIST	—	Solid	—	2.63	2.32
Ceramic frit raw mix - WP187	Keraben	—	Powder	—	0.93	0.82

To prepare the Bi4Ti3O12 sample, commercial Rutile (TiO2) (Alfa Aesar, CAS number 13463–67–13 mean particle size 1.04 μm, purity >99.9%) and μ-Bi2O3 (Aldrich, CAS number 11078–74–3 particle size 11.89μm, purity 99.9%) were used as starting materials. The appropriate stoichiometric amounts of the starting materials were mixed in an attrition-mill (in a PTFE Teflon container) with 100 g of 1.2 mm zirconia (ZrO2) balls in water for 3 hours. T5003 Rohm&Haas dispersant (0.6 wt%) was added to improve homogenization. The powders were overnight dried at 75 °C and sieved through a 0.1 mm mesh and sintered at 1200 °C for 2 hours in an Agni GmbH electrical furnace with a 3 °C/min heating rate.

Borosilicate glass (Standard Reference Glass 717 A) employed in the experiments was provided by NIST (National Institute of Standards and Technology) as a piece with dimensions 20 cm × 10 cm × 10 cm. The sample was machined with a crown drill bit with adequate dimensions to be inserted in the quartz holder (10 mm diameter, 15 mm height).

Dielectric properties of materials and their variation with the temperature were calculated by the Cavity Perturbation Method (CPM)^43^. For the dielectric properties measurement under microwave heating, the samples were prepared in a cylindrical shape (10 mm diameter, 15 mm height) and placed at the center of the microwave cavity inserted in a quartz tube.

For the dielectric properties measurement, the low power microwave source of a vectorial network analyzer (VNA) coupled at the bottom wall of the microwave cavity (see Fig. 1) generated a signal from 1.6 to 2.2 GHz and the reflected signal from the microwave cavity was continuously measured to determine the resonant frequency and quality factor variations of the cavity as a function of the temperature in the material under test (MUT). These parameters (resonant frequency and quality factor) were introduced in the CPM formulas for dielectric constant and loss factor calculations. A complete and detailed information of these calculations is given in reference^20^.

The heating rate was 5–10 °C/min depending on the sample. The microwave heating system is based on another frequency sweeping source (from 2.2 to 2.6 GHz) and a microwave amplifier that, together with an adjustable coupling device (electric probe with variable penetration) and a double directional coupler (MECA’s 722–40–1.950), allows for generating, adjusting and measuring the microwave forward and reflected signals in the microwave cavity as a function of the frequency and temperature. By selecting the appropriate frequencies of the source around the frequency peak of the cavity, we provide an excitation with a pulsed shape to supply the required microwave power depending on a desirable level of heating rate in the sample (maximum delivered power was 150 W). More detailed information has been included in supplementary information.

The infrared (IR) pyrometer (Optris CT-Laser LT) was used to measure the surface temperature of the quartz holder. Since the temperature calibration is performed through the described procedures, the emissivity was fixed to 0.97 during the experiments.

The portable Raman spectrometer (ProRaman-L-532-B1S) has a 532 nm excitation, typical linewidth of 2 cm^−1^, and maximum power of 100 mW. It was calibrated following the procedure that involves the measurement of two NIST traceable standards: silicon and acrylonitrile.

For the DTA measurements, an equipment Netzcsh model STA-409 was used. The furnace of the equipment has a temperature controller TASC 414/2 Netzsch. The samples were placed on platinum crucibles. Calcined α-Al_2_O_3_ was used as reference material. The temperature range for the measurement was 30–1250 °C, under air flux of 0.04 l/min, and the heating speed was 10 °C/min.

## Supplementary information

Supplementary Information
