# Supplementary material for: Temperature Assessment Of Microwave-Enhanced Heating Processes
Source: Sci Rep. 2019 Jul 25;9:10809. doi: 10.1038/s41598-019-47296-0 (PMC6658534; doi:10.1038/s41598-019-47296-0)
Supplement: Supplementary file 1 — Supplementary Information [file 41598_2019_47296_MOESM1_ESM.pdf]

TEMPERATURE ASSESSMENT OF MICROWAVE-ENHANCED HEATING PROCESSES

B. García-Baños[1,*], J.J. Reinosa[2], F.L. Peñaranda-Foix[1] , J.F. Fernández[2], and J.M. Catalá-Civera[1]

Additional measurements for temperature calibration

Fig. S1 shows the calibration procedure described in Section 2.2.C applied to potassium perchlorate ($KClO_4$). It consists in using selected pure salts with well-known transition temperatures and measure their dielectric properties to identify the corresponding surface temperature. Potassium perchlorate has a phase transition at a bulk temperature of 298.9±0.9ºC. This transition can be identified through the dielectric properties when the pyrometer indicated a surface temperature of 245ºC. Previous studies[A] attributed this transition to a change in the orientational order of ClO4- ions in the two phases. Since this is an ionic solid, this arrangement of one part of the structure involves a higher polarizability and hence the dielectric constant increases after the transition.

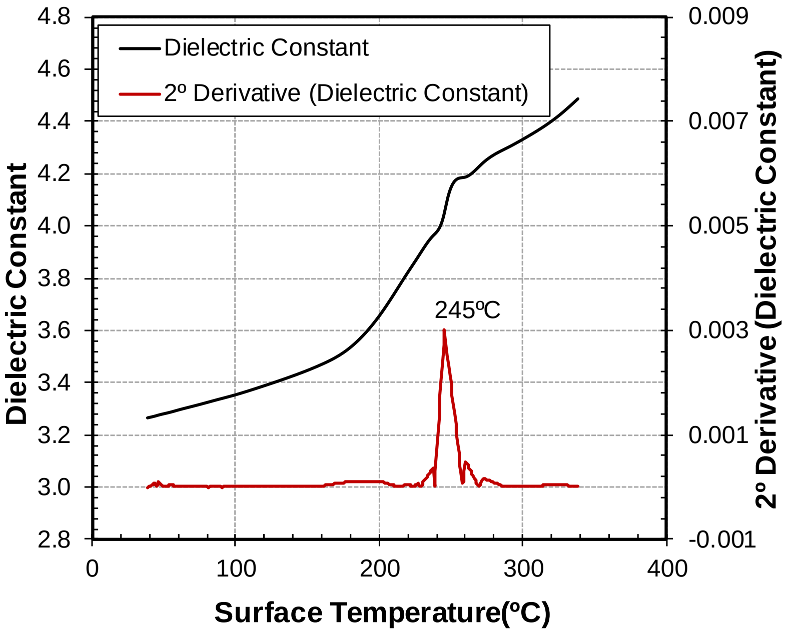

Fig. S1. Dielectric constant of potassium perchlorate as a function of the holder surface temperature measured by the pyrometer. The second derivative of the dielectric constant facilitates the identification of the temperature at which the sample changes its crystalline structure.

[A] Syal, S.K., Yoganarasimhan, S.R. Infrared and permittivity studies on alkali perchlorates. *Journal of Solid State Chemistry*, **10** (4), 332-340 (1974).

Fig. S2 shows the calibration procedure described in section 2.2.D applied to potassium sulfate (K_2SO_4). In this case, we used this reference material with a well-known Raman shift. This salt undergoes a change in its crystal structure from an orthorhombic to a hexagonal system at a bulk temperature of 585 ± 2ºC. The Raman response of the mode around 980 cm^{-1} experienced a sharp shift at a holder surface temperature of 463ºC, which was correlated with the dielectric measurements.

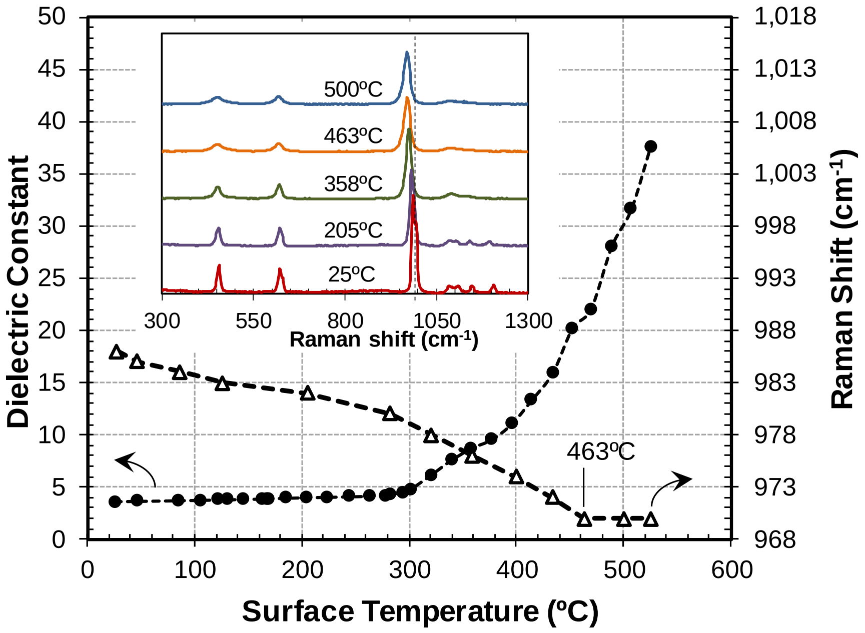

Fig. S2. Dielectric constant and Raman shift of the mode placed around 980cm^{-1} during microwave heating of a potassium sulphate sample. Inset: Raman shift spectrums obtained at different temperatures during the heating process (the dashed line shows the Raman mode of interest).

Method for dielectric properties calculation

Dielectric properties of materials and their variation with the temperature were calculated by the Cavity Perturbation Method[43] (CPM). The CPM is probably the most common technique for measuring the complex permittivity of dielectric samples at microwave frequencies.

This technique is based on the changes in the resonant frequency and quality factor in a microwave cavity due to the insertion of a sample of material under test (MUT). The main conditions for the applicability of the CPM are that the electromagnetic fields in the cavity, with and without the sample, must be very approximate. Under this assumption, the dielectric constant and the dielectric loss factor of a homogeneous and isotropic material can be directly related to the measured shift in the resonant frequency and the change in the quality factor.

The method also requires the measurement of the empty microwave cavity and the calculation of some parameters that depend on the configuration of electromagnetic fields by calibration with reference samples of the same dimensions with known dielectric properties.

For example, Fig. S3 shows the reflection factor of the microwave cavity for a porcelain sample at room temperature (25ºC) and at 680 ºC measured with the vectorial network analyzer (VNA) coupled at the bottom of the cavity. The peak and width of the reflection signal was used to determine the resonant frequency and quality factor of the cavity as a function of the temperature in the MUT and to calculate the dielectric properties with the CPM formulas.

Then, the continuous monitoring of the reflection factor of the microwave cavity with the VNA during the heating of the sample provided the measurements required for determining the dielectric properties as a function of the temperature.

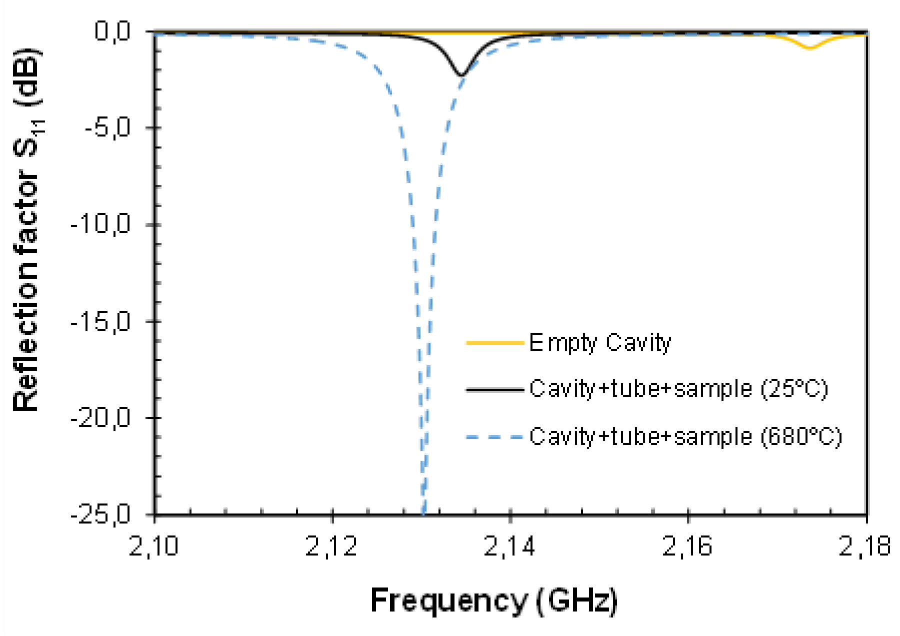

Fig S3. Reflection factor of the microwave cavity for a porcelain sample at room temperature (25ºC) and at 680 ºC measured with the low power vectorial network analyzer (VNA) coupled at the bottom of the cavity.

Method for power adjustment in the microwave heating system

The selection of the appropriate frequencies of the source around the frequency peak of the cavity provides an excitation with a pulsed shape that allows supplying the required microwave power depending on a desirable level of heating rate in the sample. Therefore, by keeping the output power of the amplifier constant to 150W, the increase or decrease of the sweep frequencies in the source results in the modification of the effective duty cycle of the pulsed excitation and hence a fast adjustment of the microwave power released to the microwave cavity.

An example of this procedure is given in the following figure. Fig S4 shows the reflection factor (S11) of the microwave cavity loaded with a quartz tube containing a sample of material (see Fig 1a) as a function of the frequency.

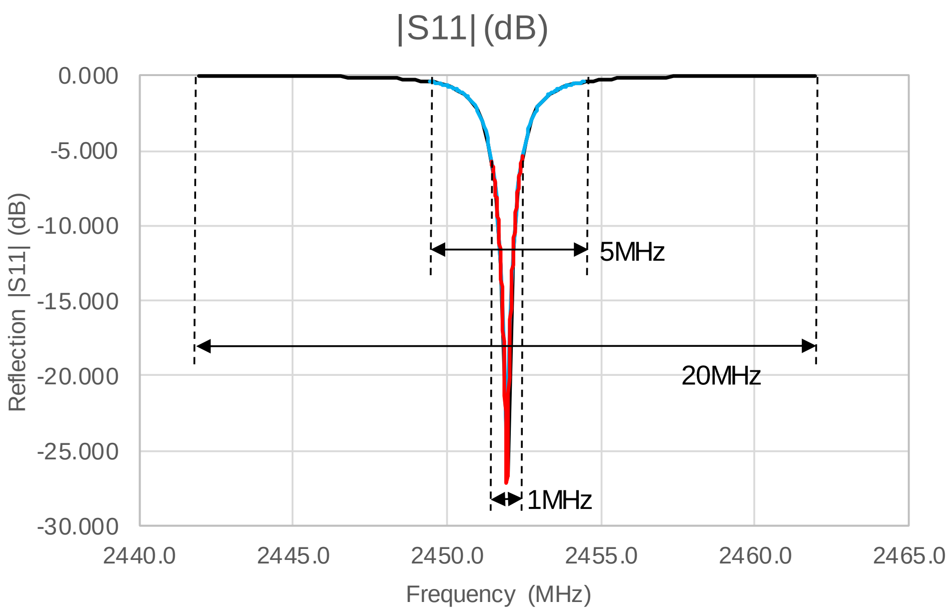

Fig S4. Reflection factor |S11| of the cavity as a function of frequency.

From Fig. S4, only microwave frequencies f_i close to the resonance peak (~2.452 GHz) are not reflected back from the cavity and contribute to heat the sample absorbing the microwave power. From the reflection factor (S11) of the cavity, the microwave absorbed power can be written as[20]:

$$P_{abs}(f_i) = P_i\left(1 - |S_{11}(f_i)|^2\right) \qquad i = 1, \ldots, N \qquad (1)$$

$$P_{aver} = \frac{1}{N}\sum_{i=1}^{N} P_{abs}(f_i) \qquad (2)$$

where $P_{abs}(f_i)$ is the absorbed power at a specific frequency f_i and P_{aver} is the average absorbed power for a frequency sweep in the range $[f_1 < f_i < f_N]$ with N frequency points.

Figure S5 illustrates the absorbed microwave power by the cavity given by eqns. (1) and (2) with 3 different frequency ranges around the frequency peak: a) 20 MHz, b) 5MHz and c) 1 MHz as a function of the sweep time (20ms per sweep with N=100 frequency points):

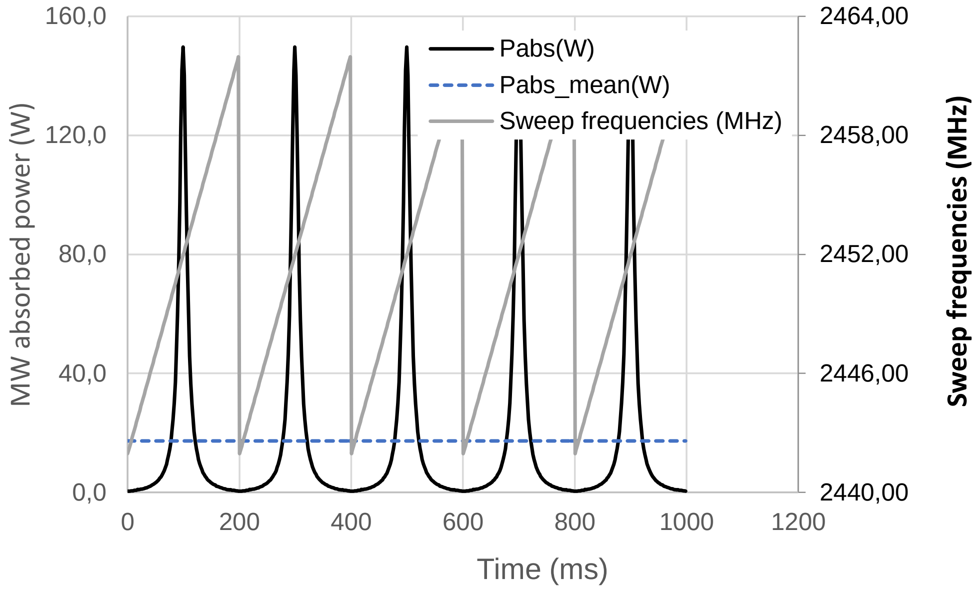

160,0
120,0
80,0
40,0
0,0
MW absorbed power (W)
0
200
400
600
800
1000
1200
Time (ms)
2464,00
2458,00
2452,00
2446,00
2440,00
Sweep frequencies (MHz)
Pabs(W)
Pabs_mean(W)
Sweep frequencies (MHz)

(a)

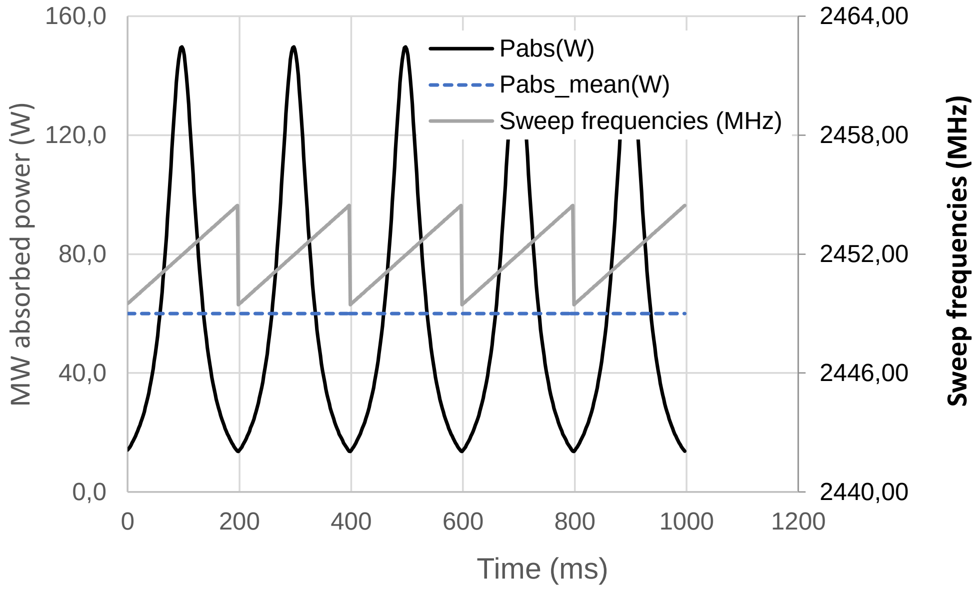

160,0
120,0
80,0
40,0
0,0
MW absorbed power (W)
0
200
400
600
800
1000
1200
Time (ms)
2464,00
2458,00
2452,00
2446,00
2440,00
Sweep frequencies (MHz)
Pabs(W)
Pabs_mean(W)
Sweep frequencies (MHz)

(b)

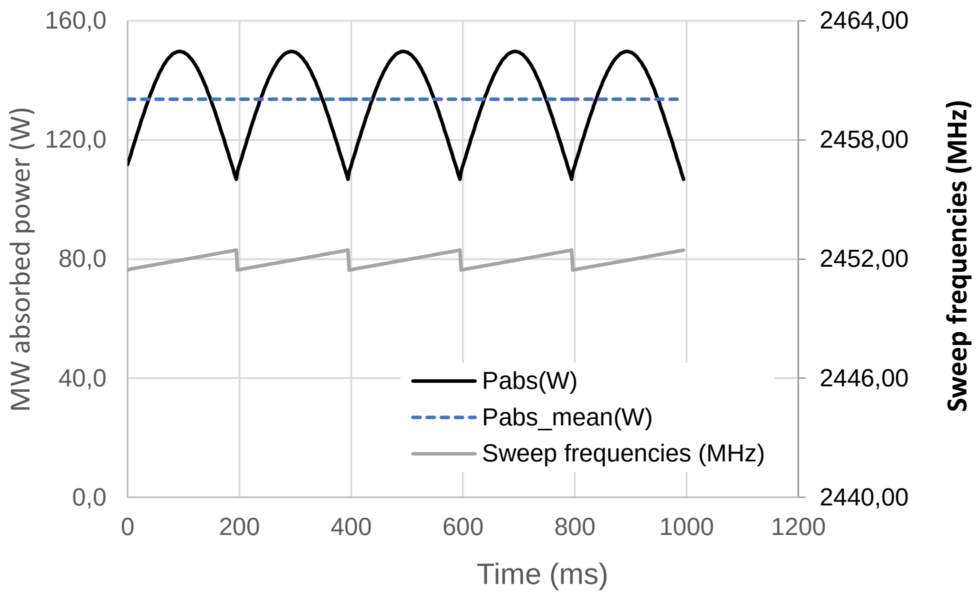

(c)

Fig. S5 Microwave absorbed power as a function of the frequency sweep of the source: a) 20MHz, b) 5 MHz, c) 1MHz. The dashed line represents the average microwave absorbed power. As the frequency sweep is narrower, the average power absorbed is higher.

Then by selecting frequency sweep ranges from (2441 to 2461 MHz – frequency sweep of 20MHz) we provide an average microwave power of 17W (duty cycle 11,3%) to the cavity. From (2449 to 2454 MHz – frequency sweep of 5MHz) we provide and effective microwave power of 60W (duty cycle 40%) and from (2451 to 2452 MHz – frequency sweep of 1MHz) we provide 134 W (duty cycle 89,3%). This procedure results in a fast adjustment of the microwave power released to the cavity which allows defining different heating rates for different dielectric materials.
